# Harnessing Artificial Intelligence to Revolutionize Microalgae Biotechnology: Unlocking Sustainable Solutions for High-Value Compounds and Carbon Neutrality

**DOI:** 10.3390/md23050184

**Published:** 2025-04-25

**Authors:** Yijian Wu, Lei Shan, Weixuan Zhao, Xue Lu

**Affiliations:** 1 Department of Fundamental Courses, Lianyungang Technical College, Lianyungang 222000, China; wuyj5131@163.com; 2 School of Information Engineering, Lianyungang Technical College, Lianyungang 222000, China; lygshanlei@126.com; 3 Institute of New Materials and Advanced Manufacturing, Beijing Academy of Science and Technology, Beijing 100089, China; zhaoweixuan@bjast.ac.cn

**Keywords:** microalgae cultivation, artificial intelligence (AI), carbon capture, biofuel production, machine learning optimization

## Abstract

Microalgae offer significant potential in diverse fields, including biofuels, carbon capture, and high-value bioproducts. However, optimizing and scaling microalgae cultivation systems present several challenges due to the dynamic interactions among environmental factors such as light intensity, temperature, pH, nutrient concentration, and CO_2_ levels, as well as species-specific biological variability. Recent advancements in artificial intelligence (AI), particularly machine learning (ML) and automation, have provided innovative solutions to these challenges. This review explored the role of AI in enhancing microalgae technology, focusing on optimizing cultivation conditions, improving CO_2_ capture, maximizing biomass production, and automating system processes. Key case studies highlight successful applications of AI in biofuel production, carbon capture projects, and high-value compound manufacturing. Key case studies demonstrate that AI-driven models can increase biomass productivity by up to 15–57%, improve CO_2_ biofixation efficiency, and enhance lipid and high-value compound yields by more than 20–43% compared to traditional methods. Additionally, we discussed the limitations of current AI models, particularly in data availability and species-specific variability, and suggested future research directions to enhance the integration of AI and microalgae systems. By leveraging AI’s potential, microalgae technologies can become more efficient, scalable, and economically viable, addressing global sustainability challenges such as energy production and climate change mitigation.

## 1. Introduction

### 1.1. Overview of Microalgae Technology

Microalgae are microscopic photosynthetic organisms thriving in diverse aquatic environments, including freshwater, marine, and brackish ecosystems. Characterized by their unicellular or multicellular structures, microalgae exhibit rapid reproductive capabilities, making them attractive candidates for various biotechnological applications. Their biological characteristics, particularly their photosynthetic efficiency, set them apart from traditional crops and other biological systems [1]. For example, some microalgae species, such as *Chlorella vulgaris* and *Arthrospira platensis*, have demonstrated photosynthetic efficiencies of up to 5–6%, significantly higher than terrestrial plants like maize (~1.5%) [2,3]. Microalgae can grow under various conditions, including autotrophic, heterotrophic, and mixotrophic modes. Autotrophic growth relies solely on light and CO_2_, making it the most common and cost-effective method for large-scale biofuel production. However, heterotrophic and mixotrophic growth modes, where organic carbon sources are added, can lead to faster growth rates and higher biomass yields, particularly under controlled conditions. Each mode presents unique advantages and challenges depending on the specific cultivation goal [4].

Microalgae are particularly promising in biofuel production. Some strains, such as *Nannochloropsis gaditana* and *C. vulgaris*, have been reported to accumulate lipids up to 50–70% of their dry weight under nitrogen deprivation or high light stress conditions [5]. These lipids can be converted into biodiesel through transesterification, offering a renewable energy source that does not compete with food crops. In addition to biodiesel, microalgal lipids can also be upgraded to green hydrocarbons through catalytic hydroprocessing, offering a pathway to drop-in fuels compatible with existing infrastructure. Research demonstrated that *Nannochloropsis* species could achieve an annual biomass production of approximately 127.8 metric ton/acre/year, outpacing traditional oilseed crops [6]. Additionally, carbohydrates in microalgae, such as starch and glucose, can serve as precursors for bioethanol through fermentation, while anaerobic digestion of whole-cell biomass produces biogas, primarily composed of methane and CO_2_. In carbon capture and utilization (CCU), microalgae provide a sustainable solution to mitigate industrial CO_2_ emissions (Table 1). Through photosynthesis, microalgae absorb CO_2_ and convert it into biomass while releasing oxygen [3]. Previous study demonstrated that microalgal cultivation in an outdoor photobioreactor removed 9–17% of industrial CO_2_ from cement flue gas, with efficiencies reaching 20–60% under optimal conditions and carbon recovery up to 10 g m^2^ d^−1^ [7]. Such integrations with industries like power generation can significantly reduce greenhouse gas emissions while producing valuable biomass. Microalgae are also a rich source of valuable bioproducts, including protein, pigments (e.g., chlorophyll, carotenoids), vitamins (e.g., B12, E), and essential fatty acids (e.g., omega-3, omega-6) [8,9,10]. For instance, *Haematococcus pluvialis*, known for its high astaxanthin content, has demonstrated production levels of up to 50 mg g^−1^ of dry biomass under stress conditions [11]. The global carotenoid market was valued at approximately USD 2.5 billion in 2024 and is expected to grow to USD 3.4 billion by 2029, fueled by the increasing demand for natural antioxidants in nutraceuticals and cosmetics [12].

Despite these advantages, several challenges hinder the widespread adoption of microalgae technology. Recent techno-economic analyses estimate production costs of microalgal biomass at USD 4–8 per kg under industrial-scale conditions, depending on the cultivation system and co-product valorization strategies employed. Unlike soybeans, microalgae can simultaneously yield lipids, proteins, pigments, and bioactive compounds, significantly enhancing economic feasibility [1,13,14]. Furthermore, the biological variability among species and the complexity of cultivation systems complicate scaling up production. Currently, there is a lack of comprehensive solutions that integrate advanced technologies like artificial intelligence (AI) to optimize microalgae cultivation and reduce production costs. Most existing approaches focus on isolated aspects of the cultivation process, failing to address the complex interplay of environmental, biological, and operational factors. An integrated, AI-driven cultivation framework offers the potential to improve biomass yield by 30–50%, reduce nutrient and energy consumption, and enhance process control accuracy for large-scale production [15,16,17].

### 1.2. The Role of Artificial Intelligence (AI)

AI is revolutionizing numerous fields, including agriculture, manufacturing, healthcare, and environmental science [18]. The transition from traditional trial-and-error approaches to AI-driven methodologies is not instantaneous and involves several key transitional tools. These tools help in systematically exploring the parameter space, developing empirical models, and scaling up processes. One such tool is the Design of Experiments (DoE), which provides a structured approach to systematically vary multiple factors and understand their interactions. DoE allows for the efficient exploration of the parameter space, leading to the identification of optimal conditions without the need for exhaustive experimentation [19]. Empirical and macroscopic modeling also play a crucial role in this transition. Empirical models, based on experimental data, provide a quantitative understanding of the relationships between process parameters and outcomes. Macroscopic models, on the other hand, capture the overall behavior of the system and can be used for process optimization and scale-up. These models serve as a bridge between traditional methods and more advanced AI-driven approaches, providing a foundation for the development of more sophisticated predictive models [20]. In the context of microalgae technology, AI plays a critical role in optimizing cultivation processes, improving product yields, and enhancing system efficiencies. By leveraging advanced algorithms and machine learning (ML) techniques, researchers and industries can address many of the inherent challenges associated with microalgae cultivation and processing.

One of the primary roles of AI in microalgae technology is the optimization of cultivation conditions. Microalgae are sensitive to a range of environmental factors, including light intensity, temperature, pH, nutrient availability, and CO_2_ concentration. Traditional cultivation methods often rely on trial-and-error approaches, which can be inefficient and time-consuming. AI-driven models, however, can analyze large datasets collected from cultivation systems to identify optimal growth conditions. For example, Yu et al. (2024) [21] developed an artificial neural network (ANN) growth model for *Synechocystis* sp. PCC 6803 based on four light regions (FLRs), achieving a validation R^2^ value of 0.97, 76.36% more accurate than traditional light–dark models. The model was validated using a separate dataset, demonstrating a prediction error of less than 5%, which highlights its robustness and reliability in optimizing microalgae growth, thereby demonstrating its effectiveness in optimizing microalgae growth. The ANN model outperforms traditional approaches by capturing complex nonlinear relationships between variables more efficiently. For example, while mechanistic models require extensive parameter tuning and assumptions, the ANN model provides a data-driven, flexible approach with significantly higher prediction accuracy and reduced error rates [22].

In addition, AI contributes to the automation of microalgae cultivation and processing systems. The integration of AI in bioreactors enables real-time monitoring and control of multiple parameters, reducing the need for manual intervention. Automation systems can operate continuously, ensuring that optimal growth conditions are maintained, which is particularly important in large-scale industrial settings. For instance, Yeh et al. (2023) [23] explored the use of ML models, including long short-term memory (LSTM) and support vector regression (SVR), for predicting the growth of *Phaeodactylum tricornutum* from outdoor cultivation. The results showed that these models outperformed traditional ones, particularly in utilizing light history data, with the LSTM model effectively capturing light acclimation effects and enabling optimization of biomass sensing and harvesting strategies. Szelag et al. (2024) [24] developed ML models to predict nutrient removal, biomass production, and photosynthetic efficiency in a pilot-scale membrane photobioreactor (MPBR) treating sewage. The study demonstrated that the multilayer perceptron (MLP) model outperformed others, enabling its use as an alternative to mechanistic models and allowing for multi-criteria optimization of operating parameters, including HRT, SRT, air flow rate, and nutrient loads.

Moreover, AI-based decision-making plays a critical role in optimizing microalgae cultivation systems. Tools such as reinforcement learning and decision trees enable real-time optimization of environmental parameters. These AI models can adjust cultivation conditions dynamically, ensuring optimal growth while minimizing resource consumption. Furthermore, integrating multi-agent systems allows for autonomous decision-making in large-scale operations, improving overall efficiency and scalability. For instance, Hossain et al. (2022b) [25] used SVR with Bayesian optimization to enhance *C. vulgaris* biomass productivity (BP) and CO_2_ biofixation (RCO_2_), achieving a high R^2^ of 0.911 and optimizing conditions to 40 °C, an N:P ratio of 1:1, and a 12/12 light–dark cycle, yielding 0.0979 g L^−1^ d^−1^ BP and 0.1408 g L^−1^ d^−1^ RCO_2_. The optimal BP and RCO_2_ values obtained were compared to those reported in several studies, with some results being higher, lower, or comparable. A similar BP of 0.092 g L^−1^ d^−1^ was reported in a study using response surface methodology (RSM), while another study observed a significantly higher CO_2_ uptake of 0.305 g L^−1^ d^−1^ in room temperature and under continuous fluorescent light (LD: 24/0) with a light intensity of 72 μmol m^−2^ s^−1^ [26,27]. The use of AI in decision-making processes not only streamlines operations but also enhances the overall effectiveness of microalgae systems in achieving sustainability goals.

Despite these advancements, challenges remain in integrating AI with microalgae technology. A major hurdle is the availability of high-quality, standardized datasets for training AI models. Additionally, the variability among microalgae species and environmental conditions requires the development of adaptable AI algorithms capable of generalizing across diverse systems. Addressing these challenges will be critical to fully realizing the potential of AI in microalgae technology. By leveraging the power of AI, microalgae systems can achieve unprecedented levels of efficiency, scalability, and economic viability. As AI technologies continue to evolve, their integration with microalgae cultivation promises transformative advancements in addressing global challenges such as energy sustainability, carbon capture, and food security.

## 2. Key Applications of AI in Microalgae

### 2.1. Optimizing Cultivation Conditions

**Figure 1:**
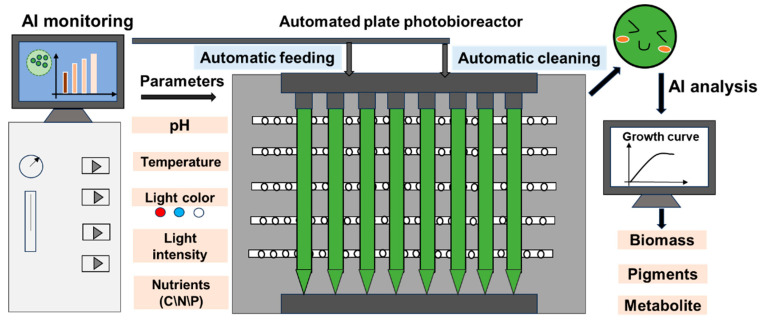
AI-guided microalgae cultivation system for biomass, pigment, and target product production.

Optimizing cultivation conditions is crucial for maximizing the productivity and efficiency of microalgae systems. Microalgae are sensitive to various environmental parameters, including light intensity, temperature, pH, nutrient concentration, and carbon dioxide availability. The interplay of these factors significantly influences their growth rates, biomass yield, and the overall quality of the produced biomass (Table 2). Traditional cultivation practices often rely on empirical methods, which can lead to suboptimal results. Advancements in technology, particularly in AI and ML, are enabling a more systematic and data-driven approach to optimizing these cultivation conditions (Figure 1).

#### 2.1.1. Light Optimization

Light is one of the most critical factors influencing microalgae growth, directly impacting photosynthetic efficiency and biomass productivity. However, optimizing light conditions is complex due to the interplay of light intensity, duration, and spectral composition. Specific variables such as light intensity (measured in µmol photons m^−2^ s^−1^), photoperiod (hours of light per day), and spectral quality (wavelengths in nm) are crucial for maximizing growth. AI has emerged as a powerful tool to tackle these challenges by developing precise light management strategies. One approach involves using AI models to predict optimal light intensities and cycles for different microalgae species. For instance, Fernández Izquierdo et al. (2024) [28] employed an ANN coupled with a genetic algorithm (ANN-GA) to optimize light conditions for polyphenol production in *Parachlorella kessleri*. The model identified white light at an intensity of 1000 lx and a 12:12 light–dark cycle as optimal, contributing to a polyphenol yield of 28 mg gallic acid equivalents per gram dry weight (GAE/g DW), highlighting the importance of tailored light parameters for metabolite enhancement. Compared with traditional models, the ANN model exhibits significant advantages. The ANN model achieved a validation R^2^ value of 0.97, with a prediction error of less than 5%. In comparison, the mechanistic model achieved a validation R^2^ value of 0.85, with a prediction error of 10%, while the hybrid model achieved a validation R^2^ value of 0.92, with a prediction error of 7%. Furthermore, the ANN model demonstrated superior computational efficiency, requiring significantly less time for training and prediction compared to the mechanistic and hybrid models. The mechanistic model required extensive computational resources for solving differential equations, while the hybrid model, although more efficient than the mechanistic model, still lagged behind the ANN model in terms of speed. Furthermore, the ANN model showed better generalization capabilities, performing well across a range of light intensities and photoperiods. The mechanistic model, while accurate within its calibration range, struggled to generalize beyond the specific conditions it was calibrated for. The hybrid model exhibited intermediate generalization capabilities, performing better than the mechanistic model but not as well as the ANN model [28].

In scaling up photobioreactor systems, computational fluid dynamics (CFDs), integrated with AI and Internet of Things (IoT) technologies, has been utilized to optimize light conditions and enhance production efficiency. For example, Tummawai et al. (2024) [29] developed a closed tubular photobioreactor equipped with sensors to monitor light intensity, temperature, and other parameters, and combined with ML models to forecast growth dynamics. A constant 24 h lighting strategy improved biomass productivity by 7.19% compared to a 12 h cycle, demonstrating the critical role of light optimization in high-efficiency microalgae systems. The eXtreme Gradient Boosting (XGBoost) model demonstrated superior accuracy in growth prediction, achieving an R^2^ of 0.9997 and significantly lower error metrics (MSE: 0.0063, RMSE: 0.0792) compared to the random forest model (MSE: 0.0604, RMSE: 0.2458), indicating a more precise fit to the training data.

#### 2.1.2. Temperature Control

Temperature is another critical parameter influencing microalgae growth and metabolic activity. Each microalgae species has a specific temperature range that maximizes growth, and deviations from this range can lead to reduced productivity or cell death. Understanding the thermal optima for different strains is essential for optimizing cultivation conditions.

AI-driven models are increasingly used to predict and maintain optimal temperature conditions. For example, Janjua et al. (2024) [30] investigated the effects of temperature on *C. vulgaris* cultivation for carbon dioxide capture, using deep neural networks (DNNs) and RSM to optimize conditions. Their study identified an optimal temperature of 29.55 °C with DNN, outperforming RSM’s prediction of 28.74 °C. The DNN model achieved a prediction accuracy of 95% with a prediction error within ±1 °C, demonstrating its precision in temperature optimization, leading to enhanced CO_2_ capture efficiency and biomass productivity, in turn emphasizing the critical role of precise temperature control in microalgae cultivation systems. Specifically, the DNN model achieved a 15% increase in biomass productivity compared to traditional temperature control methods, highlighting the significant benefits of AI-driven temperature optimization and control. Automated temperature control systems can be integrated into cultivation facilities, utilizing AI algorithms to continuously monitor and regulate temperature based on real-time data, ensuring that microalgae cultures remain within their optimal thermal ranges.

#### 2.1.3. Nutrient Management

Nutrient availability is a crucial factor influencing the growth and metabolic productivity of microalgae. Elements such as nitrogen, phosphorus, and trace metals are integral to cellular processes like photosynthesis and lipid biosynthesis. Imbalances or limitations in these nutrients can significantly reduce biomass productivity and hinder the synthesis of high-value metabolites. AI has revolutionized nutrient management by providing tools to optimize nutrient supply dynamically and efficiently [31].

AI-driven models are frequently used to balance nutrient ratios for optimal growth. For instance, Hossain et al. (2022a) [32] applied SVR combined with a GA to optimize the nitrogen–phosphorus (N:P) ratio for *Chlorella kessleri* in municipal wastewater treatment. Their model identified an optimal N:P ratio of 6:1, achieving nutrient removal efficiencies of over 93% under conditions of 29.3 °C and continuous light (24/0 h/h). The SVR model had a prediction error of less than 3%, highlighting its effectiveness in balancing nutrient ratios for optimal growth. This study highlights the effectiveness of advanced AI models in precisely balancing N:P ratios to maximize nutrient removal efficiency. Additionally, a case study on *C. vulgaris* cultivation under varying N:P molar ratios (2–67) demonstrated that nitrogen or phosphorus limitation significantly affected nutrient uptake efficiency, although biomass productivity remained relatively stable. Using GA-optimized ANN models, the study achieved high predictive accuracy (R^2^ = 0.951 for biomass productivity), highlighting the relevance of nitrogen limitation strategies for optimizing nutrient removal and informing lipid induction approaches in microalgal systems [33].

In addition to ratio optimization, IoT-enabled nitrogen, phosphorus, and potassium (NPK) sensors have proven effective in monitoring real-time nutrient composition in microalgae cultivation systems. For instance, Madhumathi et al. (2020) [34] and Sørensen et al. (2015) [35] reported the application of NPK sensors, which measure N, P, and K levels, in agricultural systems. These sensors can be adapted for microalgae cultivation to track nutrient depletion and signal operators when harvesting is optimal or when additional nutrients are needed to sustain growth. This approach enhances resource efficiency, reduces nutrient waste, and ensures consistent production, thus aligning with sustainability goals in microalgae systems.

#### 2.1.4. CO_2_ Concentration and Supply

CO_2_ is the primary carbon source for microalgae, and optimizing its supply is essential for enhancing photosynthetic efficiency and biomass yield. While microalgae can utilize atmospheric CO_2_, they often benefit from higher concentrations of CO_2_, particularly in industrial settings where flue gases are available. Kim et al. (2016) [36] developed a microcontroller-based system incorporating an MG-811 CO_2_ sensor to manage CO_2_ levels in microalgae cultivation. The sensor, highly sensitive to CO_2_ and equipped with a heating circuit, maintained concentrations above 350 ppm, thereby triggering responses at a voltage of 2.75 V. Over 35 days, algae cultivated with the microcontroller system achieved a 16.5% higher concentration than those grown under natural indoor conditions. The system was validated using a separate dataset, demonstrating a prediction accuracy of 92%, thus showcasing its effectiveness in optimizing environmental parameters for enhanced microalgae growth. Industrial flue gases typically contain 2–10% *v*/*v* CO_2_ and optimizing CO_2_ supply to these levels can significantly enhance microalgae growth rates. The composition of industrial flue gases is highly variable and may include impurities such as nitrogen oxides (NO_x_), sulfur oxides (SO_x_), ammonia (NH_3_), and volatile organic compounds (VOCs). These components can significantly affect microalgal growth, photosynthetic efficiency, and overall system design. Therefore, understanding and managing gas purity is essential for effective CO_2_ biofixation at scale.

#### 2.1.5. Integrated Systems for Cultivation Optimization

The optimization of cultivation conditions is most effective when approached holistically, integrating various environmental factors. Xu et al. (2020) [37] developed a transmission hyperspectral microscopic imager (THMI) combined with ML for microalgae cultivation optimization. The system achieved high spatial and spectral resolutions (4 µm and 3 nm), using principal component analysis (PCA) and support vector machine (SVM) to classify microalgae species with 94.4% accuracy. A random forest model predicted *Phaeocystis* growth stages with 98.1% accuracy, highlighting the potential of integrating hyperspectral imaging with ML for real-time monitoring and optimization in microalgae cultivation systems.

AI can facilitate this integration by analyzing complex datasets from multiple sensors monitoring light, temperature, nutrients, and CO_2_ levels. ML algorithms can identify patterns and interactions among these variables, allowing for the development of comprehensive cultivation strategies that maximize biomass productivity. Ariawan et al. (2018) [38] developed an IoT-based system to optimize *Arthrospira* cultivation, utilizing Arduino microcontrollers and sensors for real-time monitoring of key parameters, including water temperature, light intensity, and turbidity. The system maintained stable water temperatures at 30 °C, regulated ultraviolet light intensity, and ensured optimal turbidity levels while balancing oxygen, nitrogen, and CO_2_ supply. Such systems can be significantly enhanced by integrating ML algorithms for real-time data processing, anomaly detection, and predictive control in microalgae cultivation.

### 2.2. Improving Biomass Production

Enhancing biomass production in microalgae cultivation remains a primary goal for its applications in biofuels, pharmaceuticals, and functional foods. Recent advancements in AI, IoT, and advanced cultivation systems have provided innovative approaches to optimize environmental conditions and operational parameters, significantly boosting biomass yields.

#### 2.2.1. AI-Driven Strain Development

**Figure 2:**
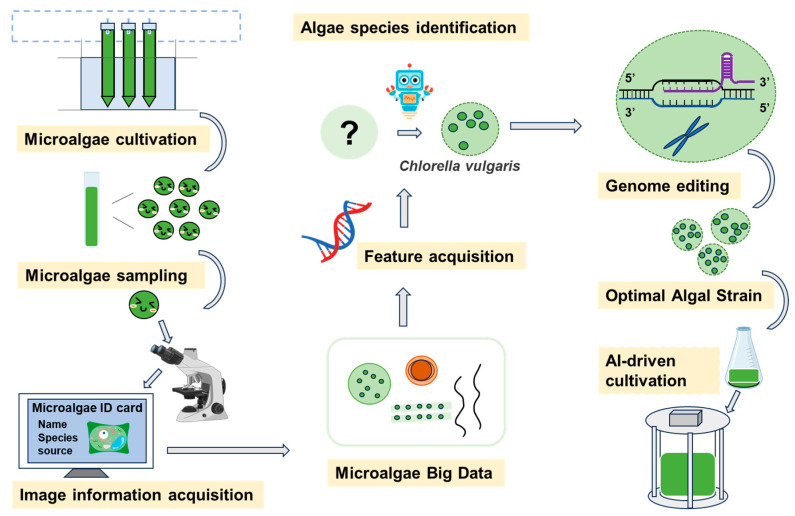
Conceptual framework integrating AI, systems engineering, and data analytics for intelligent microalgae screening, monitoring, and process control.

AI-driven approaches are transforming strain development in microalgae by providing tools to identify and enhance key traits for improved productivity (Figure 2). These methods leverage advanced ML and predictive modeling to streamline the optimization of strains for specific applications, such as biofuels and high-value metabolites. Specific tools include ANNs for predicting growth rates, GAs for optimizing nutrient uptake, and ML models for identifying genetic markers associated with high lipid content.

Heidari Baladehi et al. (2021) [39] established a reference ramanome database comprising single-cell Raman spectra (SCRS) from over 9000 cells of 27 diverse microalgal species in stationary and exponential phases. By combining pre-quenching “pigment spectrum” (PS) and post-quenching “whole spectrum” (WS) signals, species and growth states were classified with 97% accuracy using ensemble ML, providing valuable insights into strain selection. While the model achieved high accuracy in classifying species and growth states (97%), its generalization of other microalgal species may require additional calibration and validation. The approach also revealed biosynthetic profiles and metabolite interconversions at the single-cell level. Additionally, Raman-activated sorting and sequencing enabled the functional and phylogenetic characterization of uncultured microalgae. This AI-driven technique enhances strain development and genetic engineering efforts, supporting data-driven optimization of growth conditions for improved microalgae productivity. For example, the use of AI-driven models has led to the identification of specific genetic markers associated with high lipid yield in microalgae, enabling targeted genetic modifications to enhance biofuel production. Coşgun et al. (2023) [40] developed a decision tree ML model using 4670 data points from 102 published studies to investigate key factors affecting microalgal biomass and lipid production. The model identified 11 input combinations that led to high biomass productivity and 13 combinations that enhanced lipid content, demonstrating the effectiveness of ML in optimizing biofuel production conditions. Although promising, genetic engineering of microalgae remains limited by low transformation efficiency, strain-specific vectors, and complex regulatory pathways, making it a highly challenging task for most species. Panahi et al. (2019) [41] combined RNA-seq meta-analysis with supervised ML to explore the salt stress responses of *Dunaliella salina* and *Dunaliella tertiolecta*. Using Fisher’s *p*-value combination for cross-species analysis, the study identified core meta-genes linked to lipid and nitrogen metabolism, photosynthetic apparatus proteins, autophagy, and ROS-related pathways. Key interactions between Ca^2+^ signaling, lipid accumulation, and ROS networks under salt stress were also revealed. This integrative approach highlights candidate genes for genetic engineering and advances strain development for optimized metabolite production. Traditional methods often rely on trial-and-error, which can be time-consuming and less precise. In contrast, AI models provide a more systematic and data-driven approach to identifying key metabolic pathways and candidate genes for genetic modification.

#### 2.2.2. Data-Driven Optimization of Growth Conditions

AI’s ability to process and analyze large datasets allows for the optimization of growth conditions to improve CO_2_ capture. AI models can be designed to predict microalgal responses based on specific inputs such as nutrient concentrations, light intensity, temperature, and CO_2_ levels, with outputs including biomass yield, lipid accumulation, and nutrient uptake efficiency. By utilizing predictive analytics, researchers can identify the optimal combinations of these parameters to enhance growth rates and biomass yield. For instance, an ANN-GA-optimized process integrating flue gas CO_2_ sequestration and domestic wastewater utilization was developed to enhance *Scenedesmus* sp. biomass production, achieving a 57% increase in productivity under optimal conditions (light intensity: 124 μmol m^−2^ s^−1^, photoperiod: 17:7 h, temperature: 27.5 °C, pH: 9.5). The process resulted in a CO_2_ sequestration rate of 578.1 ± 23.1 mg L^−1^ d^−1^, COD reduction of 95.9 ± 2.4%, and lipid productivity of 106.4 mg L^−1^ d^−1^, with biodiesel quality meeting international standards [42].

Christian Barbosa et al. (2020) [43] developed a cost-effective sensor for real-time monitoring of microalgae biomass concentration, addressing challenges in optimizing growth conditions. The sensor, utilizing ultraviolet and near-infrared wavelengths, showed strong linearity with dry weight (DW) measurements in the range of 40–800 mg L^−1^, closely matching spectrophotometer readings. Equipped with an automatic sampling unit, the sensor operates continuously without manual intervention or sample dilution and integrates seamlessly with IoT frameworks and data loggers. This scalable solution enables precise control of growth parameters, enhancing microalgae productivity in both open and closed cultivation systems. In low-density cultures, limitations such as reduced signal strength, lower sensor sensitivity, and data sparsity can impair the accuracy and robustness of AI models, highlighting the need for improved sensing technologies and preprocessing techniques. Jia et al. (2015) [44] developed a multi-wavelength optical density sensor unit for real-time monitoring of microalgae growth, facilitating data-driven optimization of growth conditions. The system included laser diodes, photodiodes, a driver circuit, a flow cell, and a temperature-controlled housing, enabling accurate biomass estimation at 650, 685, and 780 nm wavelengths. Capable of measuring concentrations of up to 1.05 g L^−1^ (1.51 × 10^8^ cells mL^−1^) without sample preparation, the sensor provided real-time insights into culture dynamics and physiological changes. At high cell densities, self-shading reduces light penetration, impacting photosynthetic efficiency. This necessitates careful photobioreactor design and poses challenges for AI model accuracy due to increased system heterogeneity.

#### 2.2.3. Cell Harvesting and Extraction

Once microalgae reach maturity, harvesting and processing become critical steps in the overall production cycle. Automation can streamline these processes, reducing labor costs and minimizing product loss. Advanced technologies such as centrifugation, filtration, and flocculation can be integrated into automated systems to efficiently separate biomass from the culture medium. AI can optimize these harvesting techniques by analyzing the characteristics of the microalgae biomass and the specific requirements of downstream processing [38]. For example, a ML approach was used to predict the harvesting efficiency (HE) of magnetic nanoparticles (MNPs) for microalgae flocculation based on 1151 data points incorporating nanoparticle properties, microalgal traits, and operational conditions. The optimal XGBoost model achieved strong predictive performance (R^2^ = 0.932), and SHAP analysis further revealed key factors influencing HE, thus guiding the rational design of MNPs and selection of target microalgae [45]. In addition, AI models can predict the optimal harvesting time based on biomass density and nutrient levels, ensuring maximum yield and product quality. An IoT-based system has also been developed to optimize *Spirulina* cultivation and harvesting, using real-time sensors to monitor key parameters such as water temperature, ultraviolet light intensity, and turbidity [46]. The system maintained optimal growth conditions, including a stable temperature of 30 °C and balanced oxygen, nitrogen, and CO_2_ levels, while also enabling precise control of the harvesting process through real-time cell density and water quality monitoring. This approach enhanced production efficiency, standardized harvesting, and provided valuable data for further research and large-scale applications. A backpropagation neural network (BPNN) was applied to predict harvesting efficiency in a ballasted flotation based on diverse input features, including microalgal and LDM properties. To improve predictive accuracy, the BPNN model was optimized using GA and particle swarm optimization (PSO), with GA-BPNN showing the best performance (R^2^ = 0.923, MAE = 0.041). This modeling approach, further supported by SHAP analysis and experimental validation, clearly demonstrates the effectiveness of AI techniques in optimizing microalgae harvesting strategies [47].

#### 2.2.4. Challenges in Automation Implementation

While the benefits of automation in microalgae systems are significant, several challenges remain. One major challenge is the high initial investment required for automated systems, which can be a barrier for small-scale producers and startups. Additionally, the integration of AI with existing systems requires specialized knowledge and expertise, which may not be readily available in all regions or organizations. Ensuring that automation technologies are user-friendly and accessible will be crucial for widespread adoption. Furthermore, the need for continuous monitoring and maintenance of automated systems adds to the operational costs and complexity.

## 3. AI in Microalgal Biofuel Production

Biofuels derived from microalgae are gaining attention as a sustainable alternative to fossil fuels due to their high lipid content, rapid growth rates, and ability to utilize CO_2_. However, optimizing the production of microalgae-based biofuels presents several challenges, including maximizing lipid yield, optimizing cultivation conditions, and improving processing methods. The integration of AI into the biofuel production process offers innovative solutions to these challenges, enabling more efficient and scalable production methods.

### 3.1. Optimization of Lipid Production Using AI

One of the critical factors influencing the viability of microalgae as a biofuel source is the lipid content of the biomass. Various microalgae species exhibit different lipid profiles, and identifying the optimal conditions for maximizing lipid production is essential for enhancing biofuel yield. ML models have been employed to predict and optimize lipid yields by analyzing complex datasets [48]. Coşgun et al. (2021) [49] analyzed a dataset of 4670 instances from 102 published studies, using ML to evaluate the impact of cultivation and processing parameters on microalgal biomass and lipid production. By applying decision trees and association rule mining, they identified 11 combinations linked to high biomass productivity and 13 for high lipid content, demonstrating that ML can effectively determine optimal conditions for biofuel-oriented microalgae cultivation and inform future experimental planning. In addition, Muthuraj et al. (2013) [50] performed flux balance analysis (FBA) and dynamic FBA (dFBA) on *Chlorella* sp. FC2 IITG to study carbon flux distribution under light–dark cycles under photoautotrophic and heterotrophic conditions. The study revealed metabolic shifts, including the inactivity of the pentose phosphate pathway under photoautotrophic growth and elevated glycolytic flux coupled with lipid induction during the light phase. Dynamic modeling predicted consistent maintenance energy of 1.5 mmol g^−1^ DCW h^−1^, highlighting dFBA’s utility in accurately capturing metabolic dynamics for optimizing microalgae growth conditions. Further, Wu et al. (2014) [51] utilized genome-based FBA, combined with metabolomics and ^13^C-label profiling, to explore the metabolism of *Chlorella protothecoides*, a green alga with high lipid production potential for biofuels. The reconstructed metabolic network included 272 reactions, enabling simulations of phototrophic and heterotrophic growth. Optimal conditions for biomass and fatty acid production were identified, with key metabolite concentrations, including ATP and acetyl-CoA, elevated under heterotrophic conditions. Nonstationary ^13^C flux analysis revealed low photorespiration in phototrophic cells and high TCA cycle activity in heterotrophic cells. These findings provide a framework for engineering *C. protothecoides* for enhanced biofuel production. By correlating these variables with lipid yield data, AI models can predict the optimal conditions for maximizing lipid accumulation in specific microalgae strains. In addition to computational models, experimental approaches such as fed-batch cultivation have also demonstrated notable improvements in lipid yield. For instance, fed-batch cultivation of *Desmodesmus* sp. in anaerobically digested wastewater resulted in a 2.7-fold increase in biomass concentration (1.039 g L^−1^) and over a 3.1-fold increase in lipid production (261.8 mg L^−1^) compared to batch cultivation, effectively mitigating nutrient limitation and ammonia inhibition while enhancing overall productivity [52]. Similarly, mixotrophic cultivation of *Tetraselmis* sp. FTC 209, optimized using ANN and RSM, resulted in high biomass concentration (12.38 g L^−1^) and lipid productivity (173.11 mg L^−1^ day^−1^) by modeling the effects of three key medium components: glucose (organic carbon source), NaNO_3_ (primary nitrogen source), and yeast extract (a supplementary source of nitrogen, amino acids, and vitamins) [53].

### 3.2. AI-Driven Strain Selection and Genetic Engineering

The selection of high-lipid microalgae strains is crucial for efficient biofuel production. Traditional methods of strain selection can be labor-intensive and time-consuming. AI can expedite this process through data-driven approaches that identify the best candidates for biofuel production. Genome-scale reconstructions (GEMs) are developed using databases such as KEGG (Kyoto Encyclopedia of Genes and Genomes) and MetaCyc, which provide comprehensive information on metabolic pathways and enzymes from various organisms [54]. These models are commonly applied to predict biochemical and metabolic processes, employing methods like graph or topology-based analysis, flux balance analysis (FBA), kinetic modeling, dynamic reduction of unbalanced modeling (DRUM), and constraint-based modeling with energetic considerations. Parichehreh et al. (2019) [55] applied FBA to a compartmentalized metabolic network of *C. vulgaris* to optimize both specific growth rate and lipid production using experimental exchange flux data. Their model, validated by batch and fed-batch cultivation data, predicted a lipid content of 43.6% under nitrogen starvation, demonstrating FBA’s effectiveness in identifying key metabolic and environmental factors influencing lipid biosynthesis in microalgae.

By leveraging genomic and metabolomic data, AI algorithms can analyze the characteristics of various microalgae strains and predict their lipid production capabilities. Wang et al. (2012) [56] compared iTRAQ with label-free proteomics to analyze protein content in two *Chlamydomonas reinhardtii* strains, cw15 and its lipid-rich mutant, sta6, using the LTQ-Orbitrap Velos platform. The label-free method identified 896 proteins and quantified 329, while iTRAQ identified 639 proteins and quantified 124, highlighting mechanisms driving lipid production in sta6. This enables the targeted selection of strains with desirable traits for biofuel applications. Furthermore, Imam et al. (2015) [57] introduced iCre1355, an improved genome-scale metabolic model for *C. reinhardtii*, incorporating 1355 genes, 2394 reactions, and 1133 metabolites across nuclear, chloroplast, and mitochondrial genomes. The model demonstrated enhanced predictive accuracy for growth rates, lipid yields, and metabolic responses under varying conditions, including nitrogen starvation and increased light intensity. By leveraging high-resolution transcriptomics, iCre1355 accurately modeled triacylglycerol accumulation and pathway dynamics, highlighting its value in AI-driven strain selection and metabolic engineering for optimizing microalgae in industrial applications. This approach not only accelerates the strain selection process but also diversifies the genetic pool for biofuel production.

In addition to lipids, microalgal biomass contains carbohydrates that can be fermented into bioethanol or biobutanol. Whole-biomass conversion strategies, such as anaerobic digestion, offer additional routes to bioenergy in the form of biomethane or biohydrogen. These alternative pathways diversify the energy output from microalgal systems and increase process flexibility.

## 4. AI-Enhanced High-Value Products

Microalgae are not only a source of biofuels and carbon capture, but they are also a rich source of high-value products, including astaxanthin, β-carotene, lutein, phycocyanin, eicosapentaenoic acid (EPA), and docosahexaenoic acid (DHA), which are widely sought after for their nutraceutical and pharmaceutical properties [4]. The integration of AI in the production of high-value products from microalgae has the potential to optimize yield, enhance extraction processes, and accelerate product development. This section explores various case studies that illustrate the effective application of AI in enhancing the production of high-value microalgae-derived products.

### 4.1. Optimizing Cultivation Conditions for High-Value Compounds

The production of high-value compounds from microalgae, such as astaxanthin, phycocyanin, and omega-3 fatty acids, depends heavily on cultivation conditions. Factors such as light intensity, nutrient composition, and cultivation mode can significantly influence the yield of these compounds. AI technologies, particularly ML algorithms, can analyze complex datasets to identify optimal growth conditions that can maximize the production of specific high-value compounds. For example, Yang et al. (2021) [58] developed a pH-responsive fed-batch cultivation strategy, integrated with an automated photobioreactor system, to enhance *H. pluvialis* growth and astaxanthin production under mixotrophic conditions. By coupling nutrient delivery to real-time pH monitoring, the system increased biomass concentration by 90.6% and elevated astaxanthin productivity to 4.5 mg L^−1^ d^−1^, showcasing the potential of automated control for efficient microalgal bioprocesses. Makaranga et al. (2023) [59] optimized lutein production in *Chlorella saccharophila* UTEX247 through co-culturing with *Exiguobacterium* sp., thereby achieving a lutein productivity of 298.97 µg L^−1^ d^−1^—1.45 times higher than monocultures. Biomass increased 0.84-fold under optimal conditions, with metabolomics identifying 75 metabolites in the co-culture, 46 of which were unique. Key metabolites, including thiamine precursors and BCAAs, enhanced central metabolism and carbon skeleton availability, thus boosting lutein synthesis. This approach demonstrates the potential of co-culture systems for optimizing cultivation conditions to maximize high-value compound production in microalgae. In addition, Zhao et al. (2021) [60] demonstrated that melatonin (MT) treatment enhanced the coproduction of astaxanthin and lipids in *H. pluvialis*, increasing yields 1.78- and 1.3-fold, respectively. MT improved carbon flux through glycolysis and the TCA cycle, upregulated key metabolic genes, and activated the GABA shunt to balance carbon–nitrogen metabolism. The addition of linoleic acid, succinate, and GABA further boosted astaxanthin content. These examples underscore the role of AI in tailoring cultivation conditions to enhance high-value compound production. As AI models continue to improve in precision and adaptability, future applications will focus on scaling these strategies for industrial use, ensuring efficient and sustainable production of high-value compounds.

### 4.2. AI in Genetic Engineering for Enhanced Product Yield

AI has shown immense potential in the field of synthetic biology and genetic engineering, particularly for enhancing the yield of high-value compounds from microalgae. By leveraging genomic data and advanced computational techniques, researchers can identify genetic modifications that improve the production of specific metabolites [61]. For example, Feng et al. (2010) [62] investigated carbon metabolism in *Chlorobaculum tepidum* under mixotrophic conditions using metabolic flux analysis (MFA), uncovering an alternative isoleucine synthesis pathway via citramalate synthase and key CO_2_ fixation routes such as the TCA cycle and the pyruvate synthesis pathway. Pyruvate cultures exhibited higher CO_2_ fixation flux and biomass yield (YX/S = 9.2) compared to acetate cultures (YX/S = 6.4), albeit with slower growth rates. These findings emphasize the role of unique metabolic pathways like isoleucine synthesis and fixation in biomass production, providing a foundation for AI-driven metabolic engineering to optimize product yields in *C. tepidum*. In addition, Dong et al. (2006) [63] analyzed the metabolic interactions between *Phaffia rhodozyma* and *H. pluvialis* in a mixed culture, focusing on enhanced astaxanthin yields through MFA. The study revealed a 20% increase in carbon flux toward astaxanthin synthesis in *P. rhodozyma*, attributed to oxygen enrichment from *H. pluvialis* photosynthesis. Conversely, *H. pluvialis* benefited from pyruvate and CO_2_ excreted by *P. rhodozyma*, reducing carbon flux from the Calvin cycle to catabolic pathways by 33% while increasing flux to glyceraldehyde-3-phosphate by 25%. This interaction resulted in a 2.8-fold increase in carbon flux directed toward astaxanthin synthesis compared to pure cultures.

These advancements highlight the role of AI in accelerating the development of genetically optimized strains for industrial applications. As AI algorithms continue to integrate more complex datasets and improve predictive accuracy, they will further drive innovations in microalgae biotechnology, paving the way for more sustainable and efficient production systems. Furthermore, the global market for high-value products derived from microalgae is rapidly expanding, driven by increasing consumer demand for natural and sustainable products. The incorporation of AI in the production process enhances the economic viability of these products by improving yields, reducing production costs, and accelerating time-to-market.

## 5. Challenges and Future Perspectives

### 5.1. Data and Model Limitations

In the research and development of microalgae technologies, data and model limitations pose significant challenges to the implementation and optimization of AI-driven solutions. While AI offers substantial advantages for microalgae cultivation and product development, ensuring data accuracy, comprehensiveness, and representativeness is crucial for success. This section explores the limitations related to data and models, along with their implications for the future of microalgae technology.

High-quality data are fundamental for developing effective AI models. However, in the field of microalgae research, the quality and availability of data are often limited. Many microalgae experiments are conducted on a small scale, resulting in datasets that may not be sufficiently comprehensive to represent a broader range of environmental and operational conditions. This lack of representative data can lead to suboptimal performance of AI models in practical applications. Several pilot-scale systems have adopted AI-based tools for real-time monitoring, fault detection, and nutrient dosing in photobioreactors. For example, AI-enabled control systems have been deployed to regulate CO_2_ input and light regimes, improving consistency and yield in semi-automated cultivation facilities [64]. Additionally, the variability among microalgae species and environmental conditions requires the development of adaptable AI algorithms capable of generalizing across diverse systems. For instance, numerous microalgae studies focus on specific strains or experimental conditions, overlooking the diversity of environmental factors (such as temperature, light intensity, and nutrient concentrations) [54]. Consequently, the models developed may have limited predictive capability under varied conditions, thereby affecting their effectiveness in large-scale applications. Therefore, establishing large-scale, standardized datasets that encompass the characteristics of different microalgae strains and environmental conditions is essential.

Another major challenge is the integration and interoperability of data between different research institutions and projects. Due to varying experimental methods and data formats employed by different research teams, data become difficult to share and compare, thus complicating cross-study analyses. For example, some studies utilized different metrics (such as photosynthetic efficiency and carbon dioxide absorption rates) to evaluate microalgal performance [65], making it challenging to combine diverse datasets. Addressing this issue requires the establishment of unified data standards and sharing platforms, enabling researchers to more easily share and utilize data. Additionally, developing appropriate metadata standards can assist in identifying and interpreting the sources and contexts of data, thereby enhancing data availability and reliability [65].

### 5.2. Future Research Directions

As the integration of AI and microalgae technology continues to evolve, several critical research directions emerge with the promise to enhance the efficiency and applicability of this synergy (Table 3). One of the foremost areas of focus should be the development of advanced predictive models that leverage AI to simulate various environmental conditions and their impact on microalgal growth and productivity [16]. These models could utilize ML algorithms to analyze large datasets, identifying patterns and relationships that traditional modeling techniques may overlook. By incorporating real-time data from sensors and monitoring systems, researchers can refine these predictive models to provide dynamic recommendations for optimizing cultivation conditions, leading to enhanced biomass production and resource efficiency. Additionally, future research should focus on developing standardized datasets and sharing platforms to improve data availability and model generalization.

Another promising avenue for future research is the exploration of novel microalgal strains and their genetic modifications, with the aim of increasing tolerance to stressors, such as fluctuations in light intensity, temperature, and nutrient availability [66]. AI can play a pivotal role in this process by analyzing genomic data and identifying genetic traits associated with desired phenotypes. This integration of AI with synthetic biology could facilitate the rapid identification and engineering of microalgal strains that are better suited for large-scale cultivation and carbon capture applications [67].

Additionally, integrating AI into the automation of microalgae cultivation systems holds significant potential. Future research should focus on developing smart bioreactor systems that can autonomously adjust parameters such as light, pH, and nutrient concentrations in response to real-time data [68,69]. Such systems would not only reduce labor costs but also minimize human error, leading to more consistent and reliable production outcomes. The use of AI-driven automation can also enhance the scalability of microalgae technologies by enabling the monitoring and management of extensive cultivation operations across diverse geographical locations.

Moreover, there is a need for comprehensive life cycle assessments (LCAs) of microalgae-based processes that integrate artificial intelligence tools to enhance predictive modeling, uncertainty analysis, and scenario optimization. AI can assist in processing large-scale datasets, thus identifying environmental and economic trade-offs, while also recommending optimal production pathways under varying operational conditions [70,71]. Such AI-enhanced LCAs can help identify the most sustainable practices and guide policymakers in developing data-driven regulations that promote the use of microalgae for carbon capture and biofuel production, thereby supporting the transition toward more environmentally and economically viable bio-based systems. In the future, AI may be integrated with robotics and smart automation systems to enable closed-loop control of cultivation and harvesting processes. This could include automated sensor calibration, robotic biomass collection, and real-time decision-making systems for adaptive process management.

Lastly, interdisciplinary collaboration will be essential for addressing the multifaceted challenges of integrating AI into microalgae technology. Future research should encourage partnerships among scientists, engineers, and industry stakeholders to develop innovative solutions that bridge the gap between theory and practice. Collaborative efforts can foster the sharing of knowledge and resources, leading to breakthroughs that advance both microalgae cultivation and AI applications.

## 6. Conclusions

This review highlighted the transformative role of AI in microalgae biotechnology, summarizing case studies that demonstrated up to 57% improvement in biomass productivity and over 43% enhancement in lipid and high-value compound yields. Through real-time monitoring, predictive modeling, and automated control systems, AI has proven effective in optimizing cultivation parameters, enhancing CO_2_ sequestration, and streamlining harvesting and processing. While challenges such as data quality and model generalization remain, the integration of AI offers a promising pathway to achieve scalable, efficient, and economically viable microalgae-based solutions for biofuel and high-value product production. At the current stage of development, AI can be effectively applied to optimize specific cultivation parameters, monitor growth dynamics, predict yields, and support decision-making in laboratory and pilot-scale operations. As the field continues to mature, AI holds the potential to enable fully autonomous microalgae production systems through integration with robotics, smart sensors, and adaptive control technologies, ultimately supporting closed-loop and high-throughput bioprocessing across the entire value chain.

## Figures and Tables

**Table 1:** Applications of microalgae technology.

Application Area	Description	Key Benefits
Biofuels	Microalgae are converted into biodiesel and bioethanol.	Renewable energy source, lower emissions.
Carbon Capture	Microalgae absorb CO_2_ from the atmosphere or industrial emissions.	Reduces greenhouse gas concentrations.
High-Value Products	Production of nutraceuticals, pharmaceuticals, and cosmetics.	Economic value, health benefits.
Wastewater Treatment	Microalgae can treat wastewater while producing biomass.	Environmental remediation, nutrient recycling.

**Table 2:** AI techniques for optimizing microalgae cultivation.

AI Technique	Description	Application in Microalgae Cultivation
Machine Learning	Algorithms that improve through experience.	Predicting optimal growth conditions.
Neural Networks	Computational models that mimic human brain function.	Analyzing complex relationships between variables.
Genetic Algorithms	Optimization algorithms inspired by natural selection.	Strain improvement for higher productivity.
Data Mining	Extracting useful information from large datasets.	Identifying patterns in growth and productivity.

**Table 3:** Challenges and solutions in integrating AI with microalgae technology.

Challenge	Description	Proposed Solution
Data Quality and Availability	Limited access to high-quality and comprehensive datasets.	Develop standardized datasets and sharing platforms.
Model Interpretability	Difficulty understanding AI decision-making processes.	Implement explainable AI techniques.
Integration of Disparate Data	Challenges in merging data from different studies.	Establish unified data standards.
Automation and Control	Need for automated monitoring and adjustments in systems.	Develop smart bioreactor systems with real-time feedback.

## Data Availability

The data underlying the findings of this study can be obtained from the corresponding author upon reasonable request.
